# Effect of Crosslinker Concentration on Polyacrylic Acid Hydrogel for H_2_S Adsorption: Molecular Dynamic Simulation Study

**DOI:** 10.3390/polym18202468

**Published:** 2026-10-09

**Authors:** Syed Farman Ali Shah, Nurul Ekmi Rabat, Abdul Sami, Nabila Farhana Jamaludin, Khairiraihanna Johari, Siti Musliha Mat Ghani

**Affiliations:** 1 Chemical Engineering Department, Universiti Teknologi PETRONAS, Seri Iskandar 32610, Perak, Malaysia; syed_22009368@utp.edu.my (S.F.A.S.); nabila.jamaludin@utp.edu.my (N.F.J.);; 2 Chemical Engineering Department, NED University of Engineering and Technology, Karachi 75270, Pakistan

**Keywords:** H_2_S adsorption, polyacrylic acid, TEPA impregnation, molecular dynamic simulation

## Abstract

The safe and efficient removal of hydrogen sulfide (H_2_S) from natural gas is essential to prevent environmental hazards and equipment corrosion. This study investigates the influence of crosslinker concentration on the H_2_S adsorption performance of tetraethylenepentamine (TEPA) impregnated polyacrylic acid (PAA) hydrogels through a combination of experimental analysis and molecular dynamics (MD) simulations. The synthesized hydrogel samples were experimentally characterized using Fourier-Transform Infrared Spectroscopy (FTIR) to identify the functional groups. MD simulations were performed to optimize and equilibrate the molecular structures. Simulated X-ray diffraction (XRD) patterns were generated to analyze the structural characteristics of the models. Grand Canonical Monte Carlo (GCMC) simulations were used to investigate H_2_S adsorption under different temperatures and pressures. The results reveal that increasing the crosslinker concentration reduces the hydrogel’s H_2_S adsorption capacity (down to 1.5 mmol/g) by limiting available adsorption sites, restricting swelling, and negatively affecting TEPA distribution. Conversely, lower crosslinker concentrations enhance H_2_S uptake (up to 3 mmol/g) by promoting polymer chain flexibility and enabling more effective TEPA–H_2_S interactions. This integrated experimental and simulation study provides insight into the adsorption mechanisms of H_2_S, offering valuable guidance for the development of high-performance amine-impregnated polymer hydrogels for industrial gas purification applications.

## 1. Introduction

Hydrogen sulfide (H_2_S) is a highly toxic gas with a distinctive rotten egg odor. It is frequently present in natural gas. H_2_S, an extremely hazardous and harmful gas, poses significant risks to human health and adversely affects the atmospheric environment. The capture of H_2_S has remained a vital economic and environmental challenge for over a century [1]. Therefore, various methods have been explored to reduce H_2_S gas [2,3], including absorption [4], adsorption [5], and membrane technologies [6]. Among the available methods, the use of liquid amines for removing H_2_S from natural gas streams is regarded as one of the most efficient and reliable methods [7]. Despite its widespread application, this method has numerous significant problems, including equipment corrosion, amine losses, and frequent foaming issues. Adsorption methods show possibilities for effectively removing H_2_S from natural gas.

The researchers have explored various materials for H_2_S removal, including zeolites [8], activated carbons [9], silica gel [10], and metal-organic frameworks (MOFs) [11], as well as metal-free adsorbents such as porous carbon materials, covalent organic frameworks (COFs) [12], and covalent organic polymers (COPs) [13]. However, these materials face significant limitations that hinder their practical applications. For example, MOFs are structurally unstable due to the presence of numerous unsaturated metal sites, making them susceptible to degradation in acidic environments [14]. Moreover, COFs and COPs face limitations such as expensive ligands, complex production methods, and limited H_2_S adsorption capacities [15]. Their practical usage is severely limited by these inherent disadvantages. To address these limitations, new adsorbents must be designed with low cost, easy operation, and high H_2_S adsorption capacity; possible candidates include polymer aerogels [16] and polymer hydrogels [17].

Recently, polymer hydrogels have drawn attention for their ability to adsorb toxic gases such as polyacrylonitrile [18], polyvinyl alcohol [19], polyimide [20], poly(methyl methacrylate) [21], polyethylene [22] and polyacrylamide [23] compared to other conventional adsorbents because of their flexibility, less fragile structures, surface chemistry tunability, and swelling, making them suitable for efficient gas adsorption [24]. Among these polymer hydrogels, polyacrylic acid-based hydrogel [25] has received much attention due to its inherent hydrophilicity, controllable pore size, and ease of functionalization for H_2_S removal applications [26].

**Figure 1:**
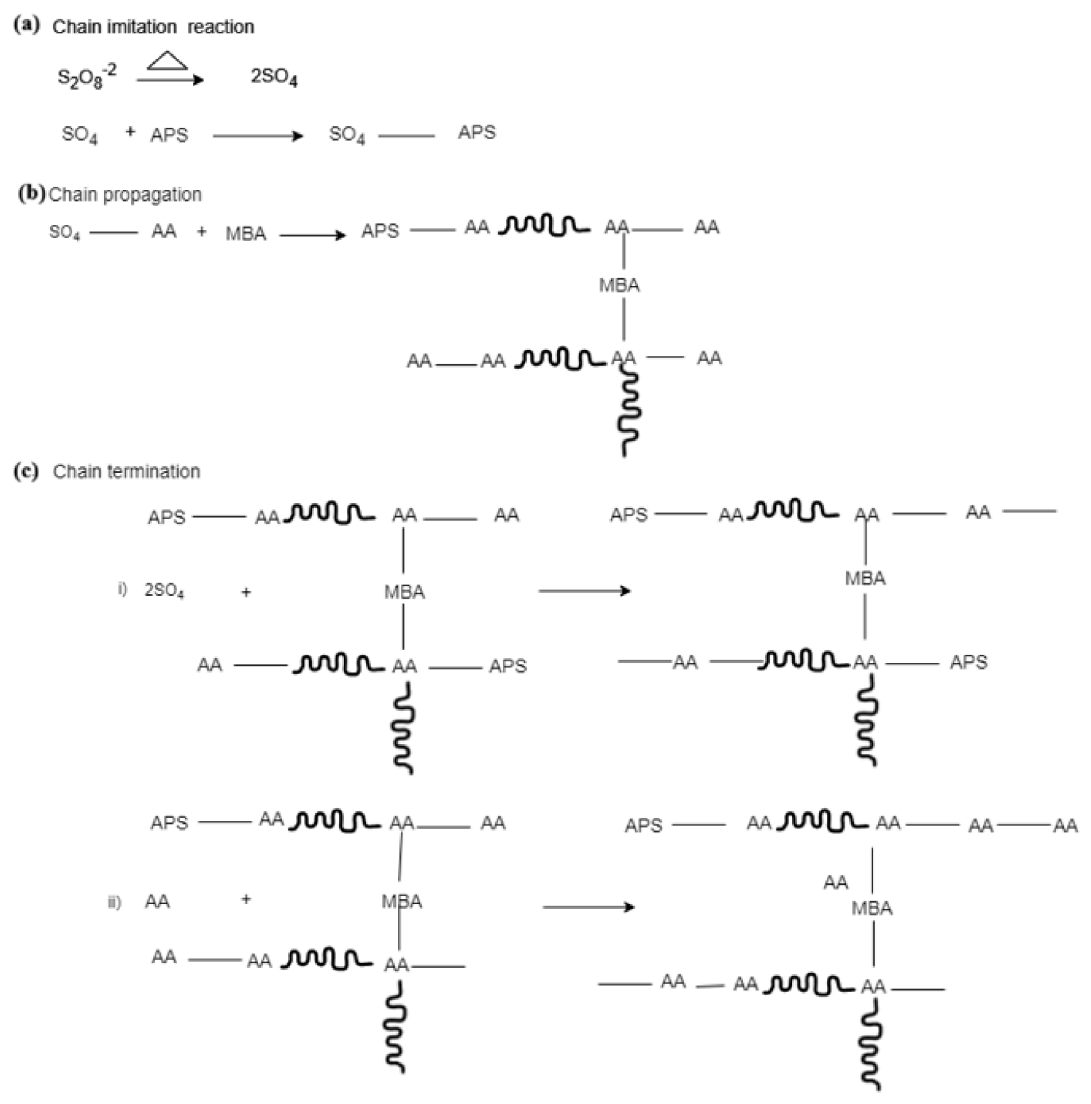
Polymer hydrogel chemical reaction mechanism (**a**) initiation (**b**) propagation and (**c**) termination of polyacrylic acid with MBA crosslinker and initiator APS.

Polymeric hydrogels are crosslinked materials produced by a simple composition of a monomer and crosslinker, as shown in Figure 1, widely used in gas separation adsorbents. The crosslinker in the hydrogels possesses the remarkable ability to absorb and retain substantial amounts of amine while maintaining its three-dimensional structure [27]. In 2020, Abdelnaby et al. [28] introduced a novel organic polymer for H_2_S adsorption from natural gas. In a recent study conducted in 2024, Liu et al. [29] explored the performance of an amine-functionalized polymer for CO_2_ capture, achieving an impressive adsorption capacity of 5.4 mmol/g at 25 °C under wet conditions. In 2023, Ghani et al. [30] reported that amine-functionalized polymer hydrogels exhibit significantly enhanced CO_2_ 0.58 mmol/g adsorption capacity compared to their unmodified counterparts. These studies have demonstrated the outstanding performance of polymer hydrogels in gas adsorption. However, most existing research has primarily concentrated on CO_2_ capture, with relatively few reports exploring its application in H_2_S [31].

Although adsorption technology is well developed, experimental handling of H_2_S gas adsorption presents significant challenges because of its hazardous nature, equipment limitations, and time constraints. To reduce the risk of human exposure, advanced computational tools [32], especially molecular dynamics simulation, are crucial for analyzing the mechanisms and interactions between H_2_S and amine-functionalized sorbents at the atomic level. Previously, a reasonable number of molecular simulation studies have been conducted on polymer hydrogels using computational tools for adsorption [33]. Molecular dynamics (MD) simulations have become a valuable tool for predicting adsorbent properties and addressing this problem. Several studies have demonstrated that the material studio (MS) software (BIOVIA Material Studio software) can be used to construct novel hydrogels for analyzing adsorption mechanisms [34].

In 2021, Amouzad Khalili et al. [35] investigated H_2_S adsorption using Grand Canonical Monte Carlo (GCMC) simulations. Their findings demonstrated a strong agreement between the simulation results and experimental data on gas adsorption. Previous studies by Farman et al. [31] investigated H_2_S adsorption using MEA-functionalized adsorbents, achieving a maximum adsorption capacity of 2 mmol/g. Their work was also validated through molecular dynamics simulations, which supported the experimental findings. In another study, MD simulations were employed to compare experimental findings with zeolite adsorbent simulations [36]. In particular, GCMC and MD simulations can be used to investigate gas adsorption at the molecular level and to complement experimental studies [37]. Therefore, in the present study, these computational approaches were employed to investigate the molecular structure and H_2_S adsorption behavior of TEPA-impregnated PAA hydrogel.

In this study, we investigate the potential of a TEPA-impregnated PAA hydrogel as an effective adsorbent for H_2_S capture. The work addresses the scientific need for environmentally friendly, amine-based polymer hydrogels that combine high adsorption capacity with a simple and scalable preparation technique. Different MBA crosslinker concentrations (1–5 wt%) were investigated to understand their influence on the polymer network, TEPA uptake, and H_2_S adsorption behavior. Molecular dynamics simulations are employed to gain molecular-level insight into TEPA–H_2_S interactions and to support understanding of the adsorption mechanism. Overall, this study aims to demonstrate the applicability of amine-functionalized hydrogels as sustainable and efficient materials for H_2_S removal.

## 2. Experimental Methodology

### 2.1. Material

Merck, in analytical reagent grade, provided a 99% pure acrylic acid (AA) monomer. Ammonium peroxide sulfate (APS) was used in the experiments, and Sigma-Aldrich (St. Louis, MO, USA) supplied 99% pure N, N′-methylene bis acrylamide (MBA) and Tetra ethylenepentamine (TEPA). All the reagents were used as received without any further purification. In this study, the polymerization process was carried out using deionized water.

### 2.2. Synthesis of Amine-Functionalized Hydrogel

**Figure 2:**
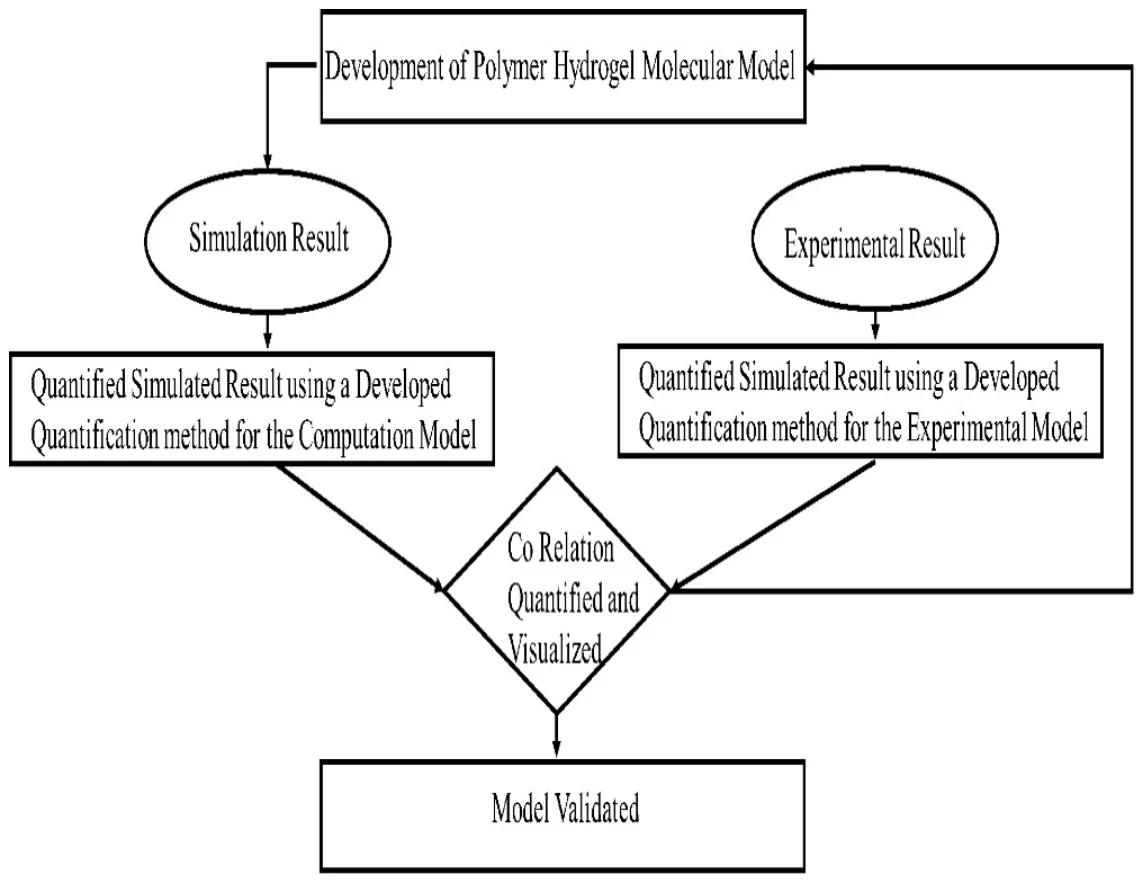
The flow diagram of the research methodology.

The polymer hydrogel was synthesized through in situ free-radical chain polymerization. A solution containing 20 g of water, 3 g of AAc monomer, and Span 80 (20% of the organic phase by weight) was prepared in a round-bottom flask and stirred for 20 min. Subsequently, MBA was added as a crosslinker at five different concentrations (1, 2, 3, 4, and 5 wt.% relative to the AAc monomer), while APS was maintained at 2 wt.% of the monomer. Paraffin oil was then added, and the reaction mixture was stirred at 500 rpm and 65 °C under the specified polymerization conditions [38]. The paraffin served as a porosity template during the free radical polymerization, which occurred within 5–10 min. The resulting polymer hydrogel was carefully removed. To eliminate residual impurities, the hydrogel was subjected to Soxhlet extraction with toluene for 24 h, followed by an acetone extraction step. A porous polymer hydrogel structure was obtained through a freeze-drying process. The flow chart methodology is described in Figure 2.

**Figure 3:**
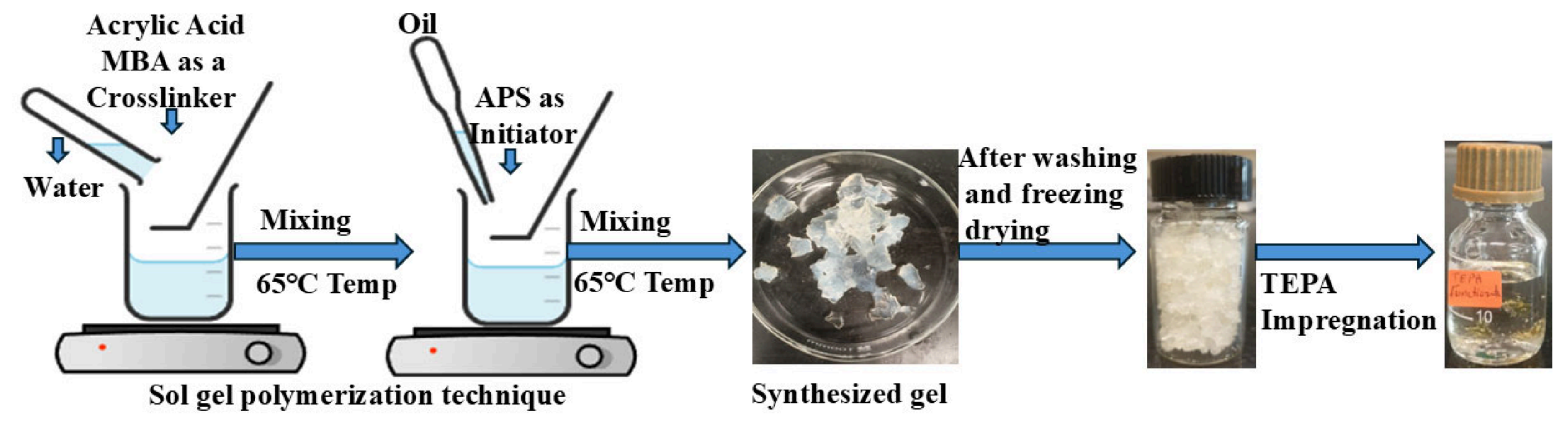
Synthesis of pure PAA with different crosslinker concentration (1–5) and TEPA amine impregnation.

To synthesize TEPA-impregnated hydrogels, 30 wt.% TEPA concentration was dissolved in 100 mL of deionized water and stirred briefly to ensure complete dissolution. The dried plain hydrogel was then introduced into the solution, and the mixture was stirred continuously for 12 h to achieve equilibrium absorption. The final product was the TEPA-impregnated hydrogel. The detailed step-by-step procedure is illustrated in Figure 3.

### 2.3. Characterization

The chemical bonding and structural properties of the different crosslinker wt.% of PAA hydrogel and TEPA-impregnated hydrogel were studied using Fourier-Transform Infrared (FTIR) spectroscopy with a Perkin–Elmer Spectrum model (PerkinElmer, Waltham, MA, USA). The FTIR spectra were collected in the frequency range of 400 to 4000 cm^−1^ using the transmission method, with sample preparation involving a mixture of the polymer hydrogel and potassium bromide (KBr). The crystallinity index (CrI) of the plain and TEPA-impregnated amine hydrogels was analyzed using an X-ray diffractometer (MiniFlex 300/600). The analysis was performed in the phase scan mode at a 2θ angle, utilizing a radiation source with a wavelength of 0.154 nm. The swelling behavior of the synthesized PAA hydrogels with different wt.% crosslinker concentrations, impregnated with 30 wt.% TEPA amine solution, were assessed by evaluating their TEPA uptake. In this analysis, dried hydrogel samples (W_i_) were immersed in TEPA solutions of 30 wt.% concentrations. The swollen hydrogels (W_f_) were removed at hourly intervals, weighed, and monitored until their weight reached equilibrium, as shown in Equation 1.

(1) $$Uptake\left(\frac{gram\;TEPA}{gram,hydrogel}\right)\%=\left(\frac{Wf-Wi}{Wi}\right)\times 100$$

### 2.4. Computational Model and Simulation Detail

#### 2.4.1. Polymer Hydrogel Construction

**Figure 4:**
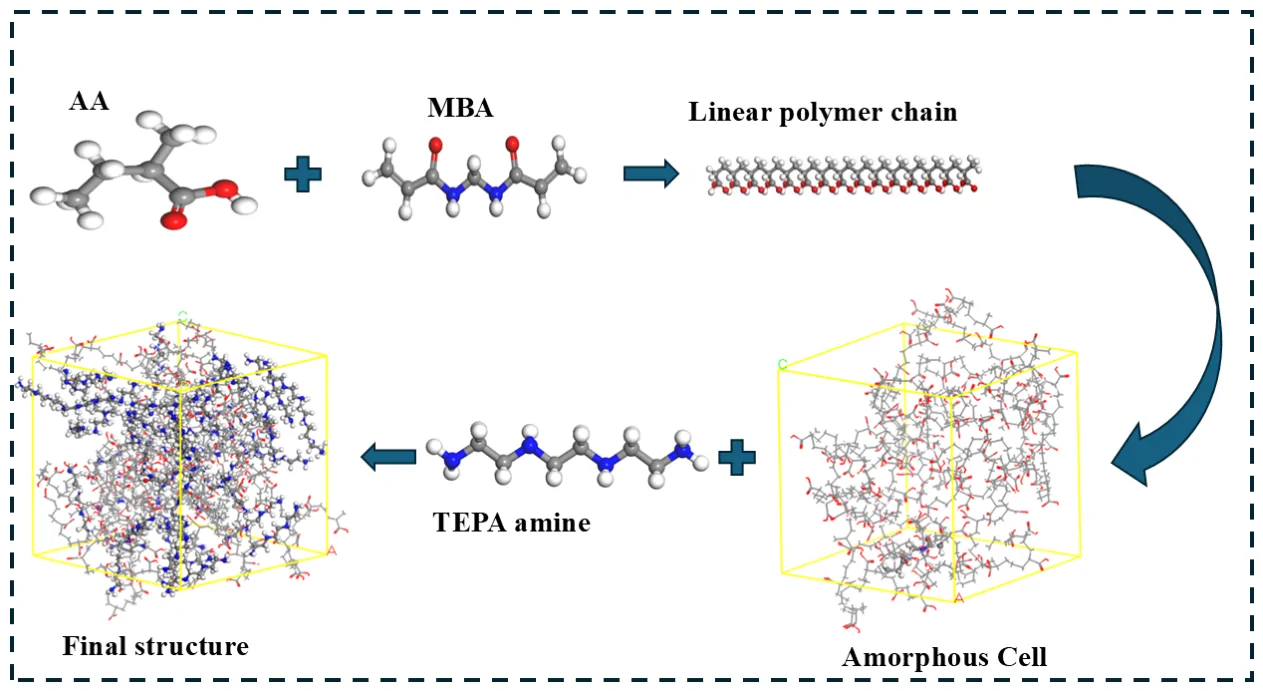
Detailed procedure for polymer hydrogel material and amorphous cell construction.

Molecular dynamics (MD) simulation is a computational method employed to ascertain the physical movements of atoms and molecules, facilitating the assessment of polymer hydrogel characteristics at the molecular level. In this study, atomistic simulations were conducted using the “Amorphous Cell” and “Forcite” modules of Materials Studio 2020 version. The initial step in conducting MD simulation using MS software (Version 2020, BIOVIA) involves constructing two polymers with repeated units: plain PAA and plain PAA functionalized with TEPA amine, as illustrated in Figure 4, following the method described by Durban et al. [39]. In the second step, the polymer hydrogel was constructed using the Amorphous Cell and Forcite modules. The initial density was intentionally selected as low, at 1 g/cm^3^, to allow the model to undergo energy minimization and structural relaxation during MD simulation, resulting in a more realistic hydrogel structure. Based on the experimental results, the density of polyacrylic acid hydrogels is in the range 1.0–1.1 g/cm^3^, depending on the degree of polymerization. The third stage involved a series of MD simulations following initial geometry optimization. First, a 500 ps MD simulation was performed in the NVT ensemble at 298 K to relax structural stresses. The lowest-energy structures were identified using a convergence tolerance of 1 × 10^−4^ kcal/mol and a displacement criterion of 5 × 10^−5^ Å. This was followed by a 200 ps MD simulation under the NPT ensemble (1 bar, 298 K) to compact the polymer hydrogel to its realistic density [40]. A thermal annealing process was then applied, consisting of five heating–cooling cycles between 298 K and 498 K in 50 K increments, with each temperature step equilibrated for 20 ps under the NVT ensemble. Finally, the geometries of the plain PAA and functionalized polymer hydrogel models were refined using an advanced minimization protocol over 10,000 iterations to eliminate unfavorable interactions and obtain fully energy-minimized configurations.

#### 2.4.2. Molecular Dynamics Simulation Details

The Grand Canonical Monte Carlo (GCMC) simulation method in MS software was utilized to examine the adsorption behavior of H_2_S within a polymer hydrogel matrix. The COMPASS II force field is employed to model atomic interactions. For this, both the Ewald summation and van der Waals interactions were determined as long-range and short-range interactions. The periodic boundary conditions are applied in the X and Y directions [34]. The simulation adsorption is conducted across a range of pressures, 0.001 to 100 KPa, and at a constant temperature of 298 K. Each simulation involves a total of 3,000,000 steps. The initial 1,000,000 steps are used to achieve system equilibration, while the remaining 2,000,000 steps are dedicated to collecting data for statistical analysis. The primary focus of this study is to confirm the adsorption of H_2_S by analyzing its diffusivity and solubility using MD simulation. The diffusivity was calculated using the Einstein relation, as given in Equation (2).

(2) $$D_{self}=\frac{1}{6N}+\sum_{t=1}^{N}|r_{i}\;(t)-r_{i}(0){|}^{2}$$

where D_self_ is the self-diffusion coefficient of H_2_S, measured in m^2^/s. N represents the total number of H_2_S molecules. N_t_ is the average number of molecular dynamics steps. t denotes the simulation time, measured in picoseconds (ps). r_i_ (0) is the displacement of the center of mass of the i-th particle at the initial time, expressed in nanometers (nm). r_i_ (t) is the position of the center of mass of the i-th particle at time t, also in nanometers (nm).

The adsorption of H_2_S was calculated by the solubility of gas molecules in the polymer hydrogel. The solubility of a gas in an equilibrated gas-polymer hydrogel system is expressed as the ratio of the gas concentration (C measured in cm^3^ (STP), polymer hydrogel per cm^3^) to the partial pressure (for mixed gas systems) of the gas i. Therefore, the solubility (S_i_) of a gas in the polymer hydrogel can be determined using the equation below (3).

(3) $$S_{i}=\underset{p\to 0}{\lim}\;(C_{i}/P_{i})$$

The conversion principle for the saturated adsorption of H_2_S, expressed in mmol/g, is determined using the Sorption module. It represents the number of H_2_S gas molecules adsorbed per unit cell (u.c.), with the original unit given as molecules/u.c. The commonly used unit, mmol/g, is calculated based on Equation (4).

(4) $$1\frac{molecular}{u.c}=\frac{10^{3}}{N\times M}=\frac{N_{ads}}{N_{A}}\times \frac{1000}{m_{sample}}=mmol/g$$

where N_A_ is Avogadro’s constant (6.02 × 10^23^), m is the unit mass, and g is the sample.

## 3. Results and Discussion 

### 3.1. FTIR Analysis

**Figure 5:**
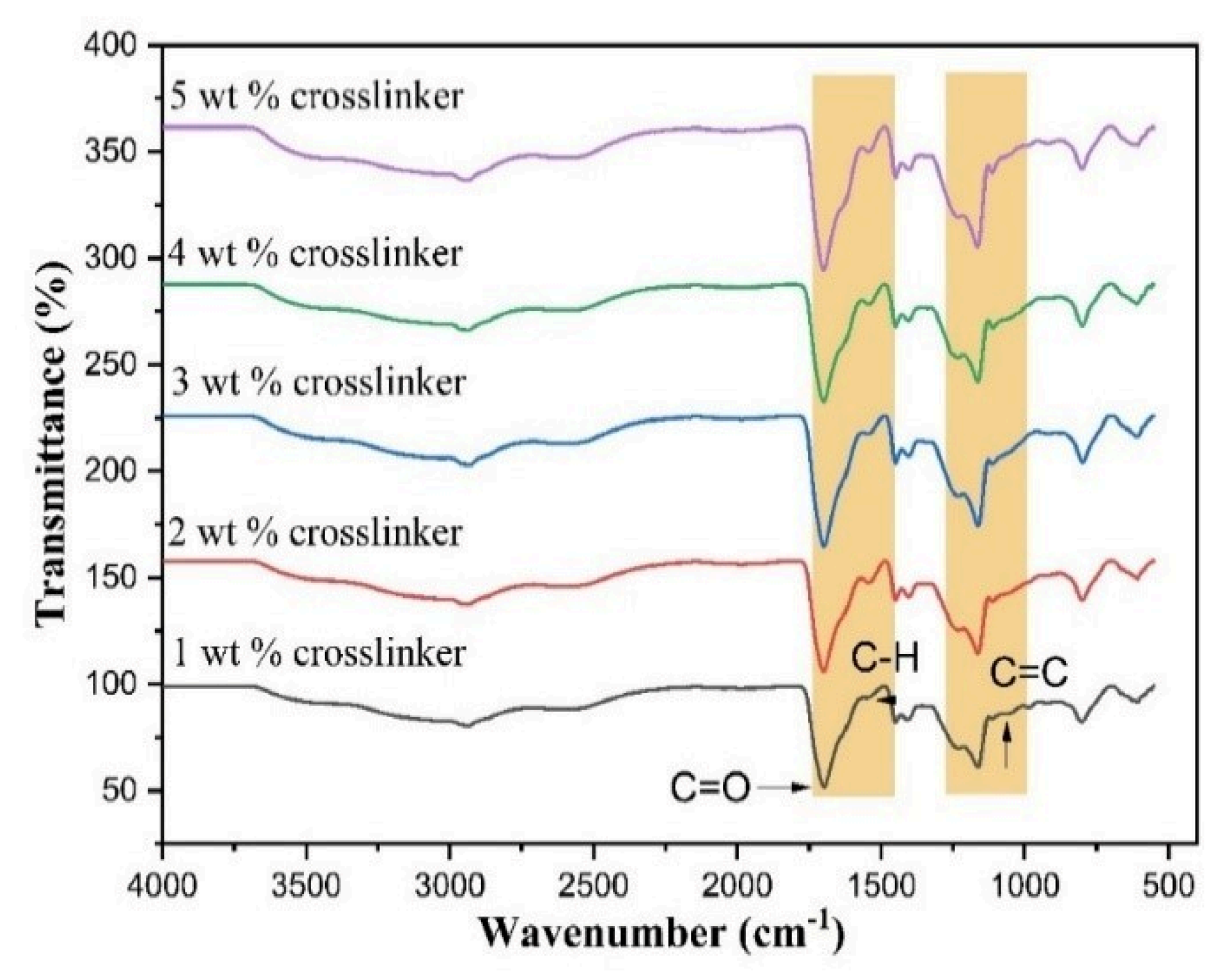
The FTIR results for pure PAA at different concentrations of crosslinker (1 to 5 wt.%).

**Figure 6:**
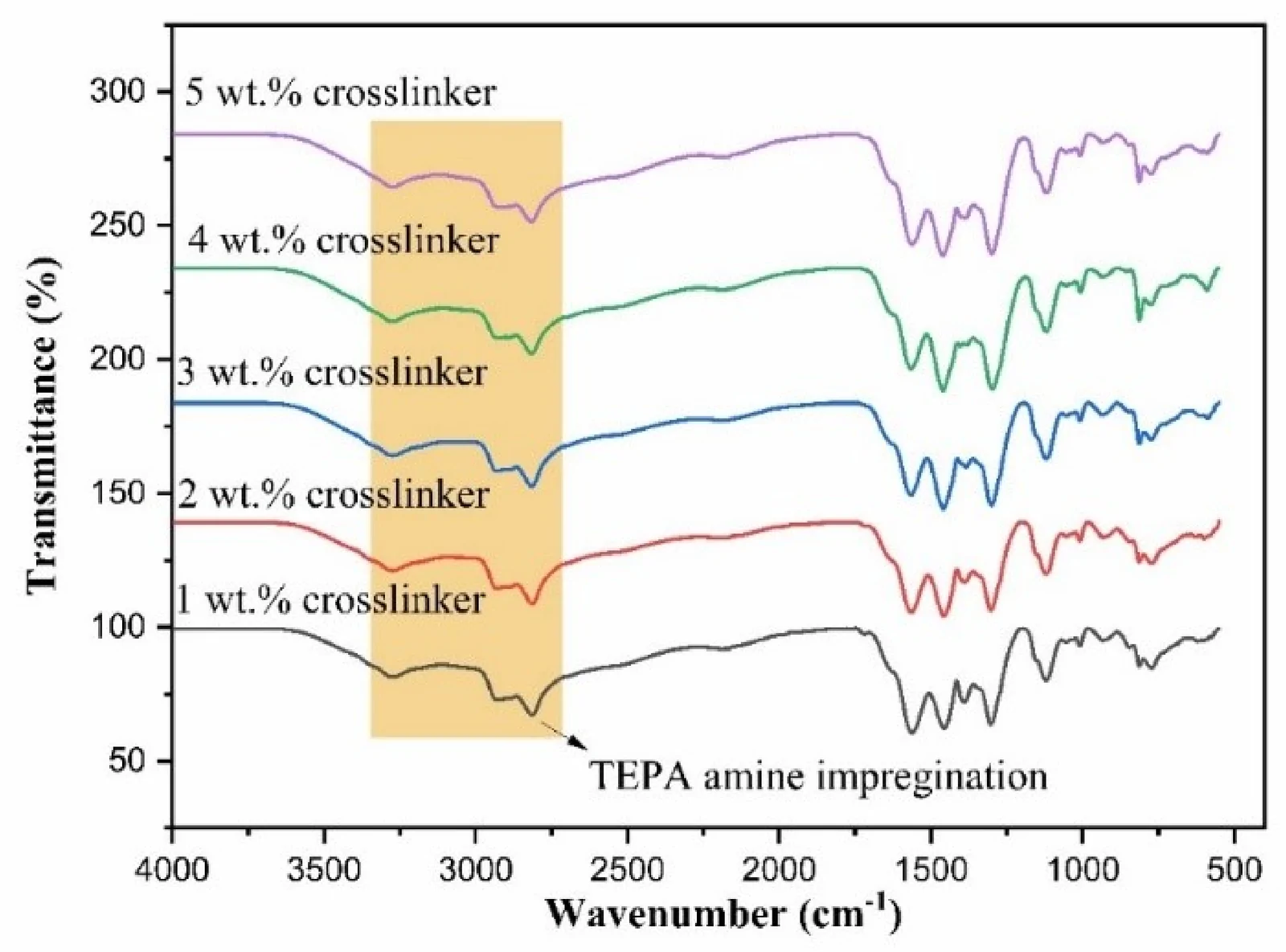
The FTIR results with the impregnation of TEPA amine in pure hydrogel.

FTIR analyses were conducted on a plain polymer hydrogel with different crosslinker ranges, 1 to 5wt.%, as mentioned in Figure 5. The obtained FTIR spectra for all the samples exhibited the expected profiles, consistent with those reported in the literature [41]. The stretching amide at the 1584 and 1090 range shows a slight increase with the rising crosslinker wt.%. This indicates a potential enhancement of the polymer network structure. The IR spectra of all samples displayed a band at 2920 cm^−1^, corresponding to C-H stretching, as reported by A. Sami et al. [16]. Figure 6 highlights the successful functionalization of TEPA on the pure PAA hydrogel, evidenced by the peak at 2945 cm^−1^. The broad band observed at 3300 cm^−1^, associated with the primary -NH- group, disappeared after TEPA amine functionalization. This disappearance is attributed to interactions with the adjacent secondary -NH- group in pure PAA, as previously documented by Ghani et al. [30]. The presence of the O=C-O- a functional group of the AAc unit was also identified at 1187 cm^−1^.

### 3.2. TEPA Amine Uptake Analysis

**Figure 7:**
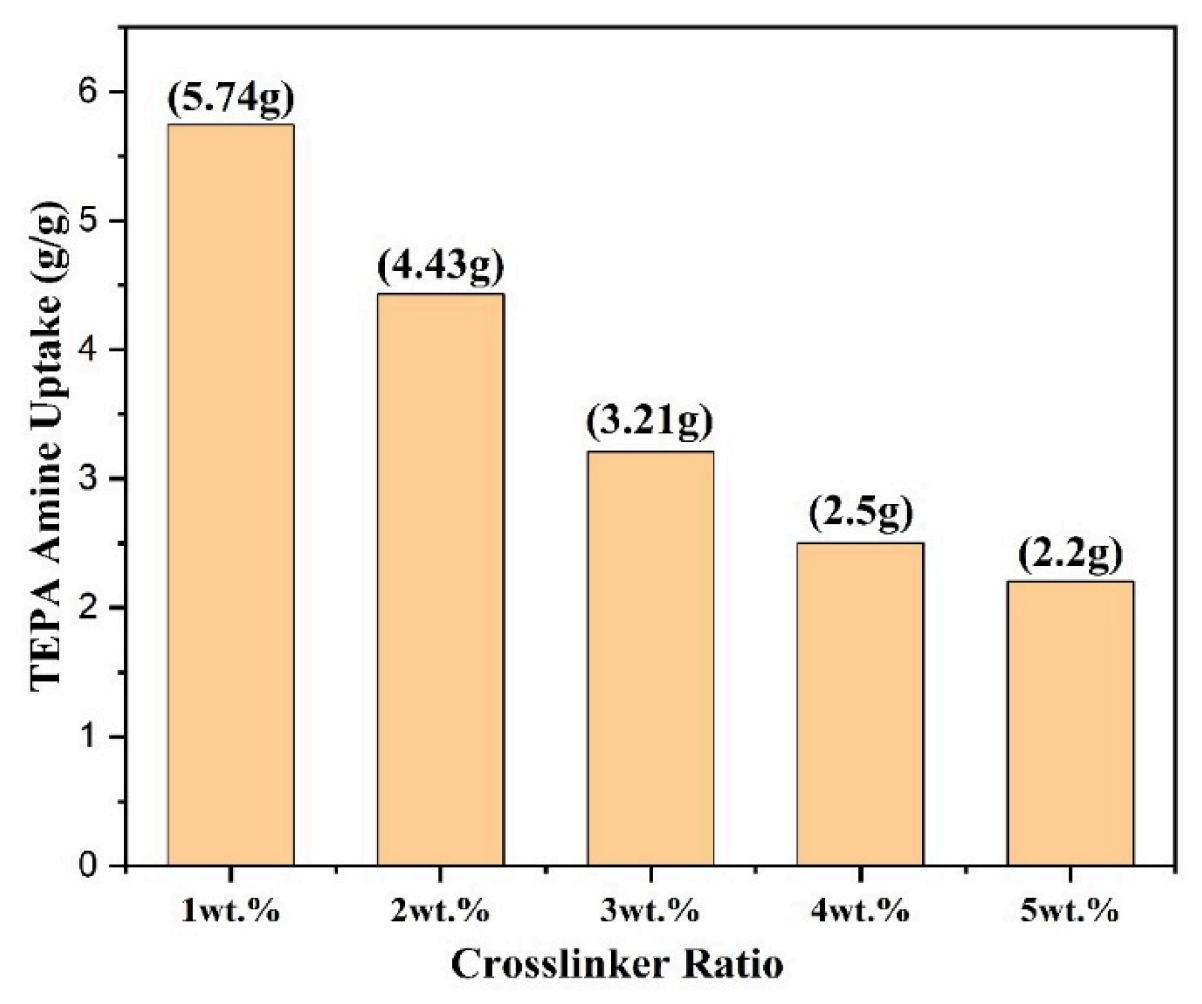
TEPA amine uptake analysis of pure PAA with different crosslinker concentrations.

Figure 7 illustrates the effect of MBA crosslinker concentration (1–5 wt%) on the uptake of a 30 wt% aqueous TEPA solution by the hydrogel at room temperature. The 30 wt% TEPA concentration was selected based on our previous study, in which different amine concentrations were investigated and the 30 wt% formulation showed the highest uptake/performance [42]. In the present study, 0.5 g of dry hydrogel was used for the uptake experiment, and the amount of TEPA-containing solution retained by the hydrogel was determined from the change in mass after impregnation. The solution uptake decreased from 5.74 to 2.20 g/g as the MBA concentration increased from 1 to 5 wt%. This decrease can be attributed to the increase in crosslinking density, which produces a more compact polymer network and restricts the swelling of the hydrogel. The increased crosslinking density limits polymer-chain mobility and reduces the available free volume for penetration and retention of the TEPA-containing solution. Consequently, higher MBA concentrations result in lower solution uptake. It should be noted that the measured values represent the uptake of the aqueous TEPA solution and therefore include both TEPA and water; they should not be interpreted as the direct dry TEPA loading of the hydrogel.

### 3.3. Simulation Results Analysis

#### 3.3.1. X-Ray Diffraction (XRD) Simulation Study

**Figure 8:**
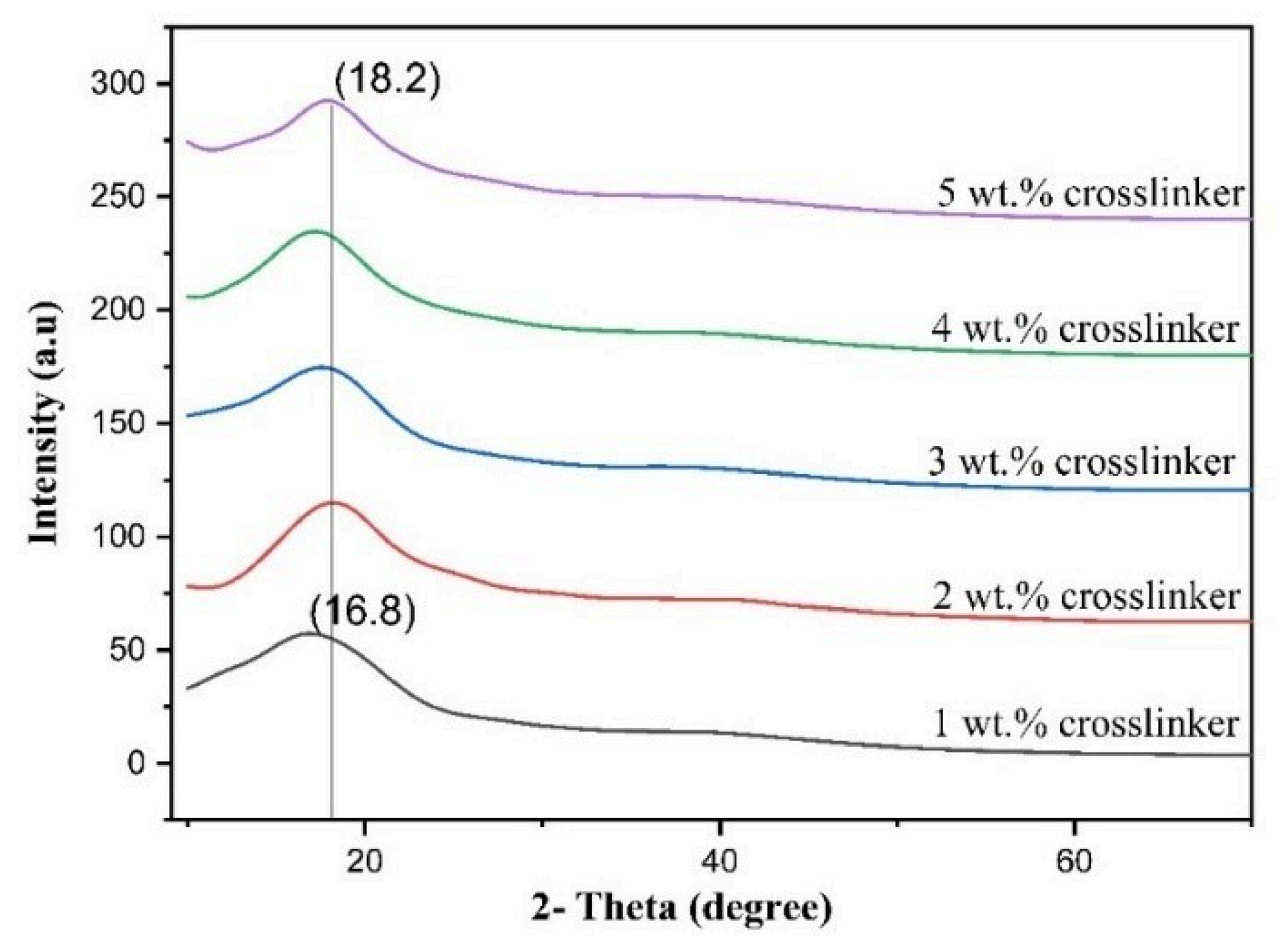
Simulation X-ray diffraction analysis of pure PAA with different crosslinker concentrations.

**Figure 9:**
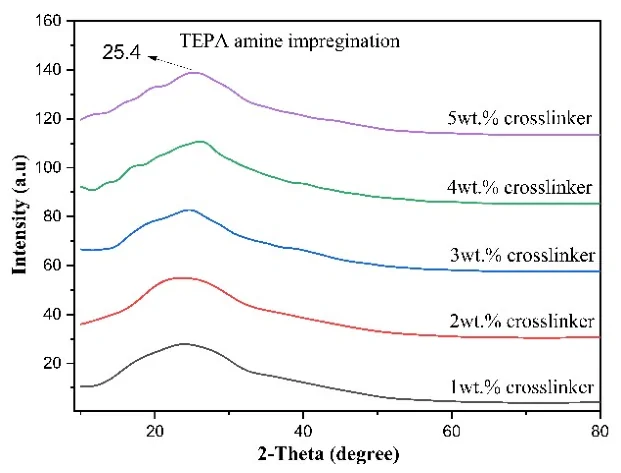
X-ray diffraction analysis of pure PAA with TEPA amine impregnation.

The XRD patterns of pure PAA and TEPA-amine-impregnated samples with varying crosslinker ratios are shown Figure 8 and Figure 9. These patterns provide insight into the structural changes induced by crosslinking and amine incorporation within the polymer network, analyzed using the force scattering module. The computed patterns, derived from the NPT trajectory, were obtained using Material Studio software. The application of the NPT ensemble ensured that the simulated patterns accurately represented the equilibrium structure under constant pressure and temperature conditions. In this study, the 2θ range varied from 5° to 50°, revealing distinct peaks for the PAA hydrogel and the TEPA amine impregnation hydrogel. Figure 8 displays the effect of different crosslinker concentrations (1–5 wt.%) on the polymer structure. The observed peaks around 16.8° and 18.2° (2θ) indicate the amorphous nature of the polymer hydrogel. The finding of this is compared with the previously published work by Luo et al. [43], who found that pure PAA hydrogel shows a peak of 18°. The shift in peak position and intensity in the molecular arrangement and network structure result from the increased crosslinker concentration. The higher intensity at 5 wt.% crosslinker implies a more ordered arrangement or stronger molecular interactions within the polymer network.

In Figure 9, a new diffraction peak emerges at 25.4° (2θ) after TEPA amine impregnation, signifying structural modifications due to the incorporation of amine groups. This change suggests that the amine molecules interact with the polymer network, potentially altering its molecular arrangement and broadening the peak. The higher crosslinker content further reinforces these structural changes, as evident from the increased peak intensity. A similar trend was also observed by Erani et al. [44], where TEPA amine is modified in synthetic material. It is proposed that the TEPA peak contributes to the increased broadness and intensity of the MCNTs/TEPA peak. This interaction likely results in increased electronic or structural modifications, leading to a more pronounced peak.

#### 3.3.2. H_2_S Adsorption Study

**Figure 10:**
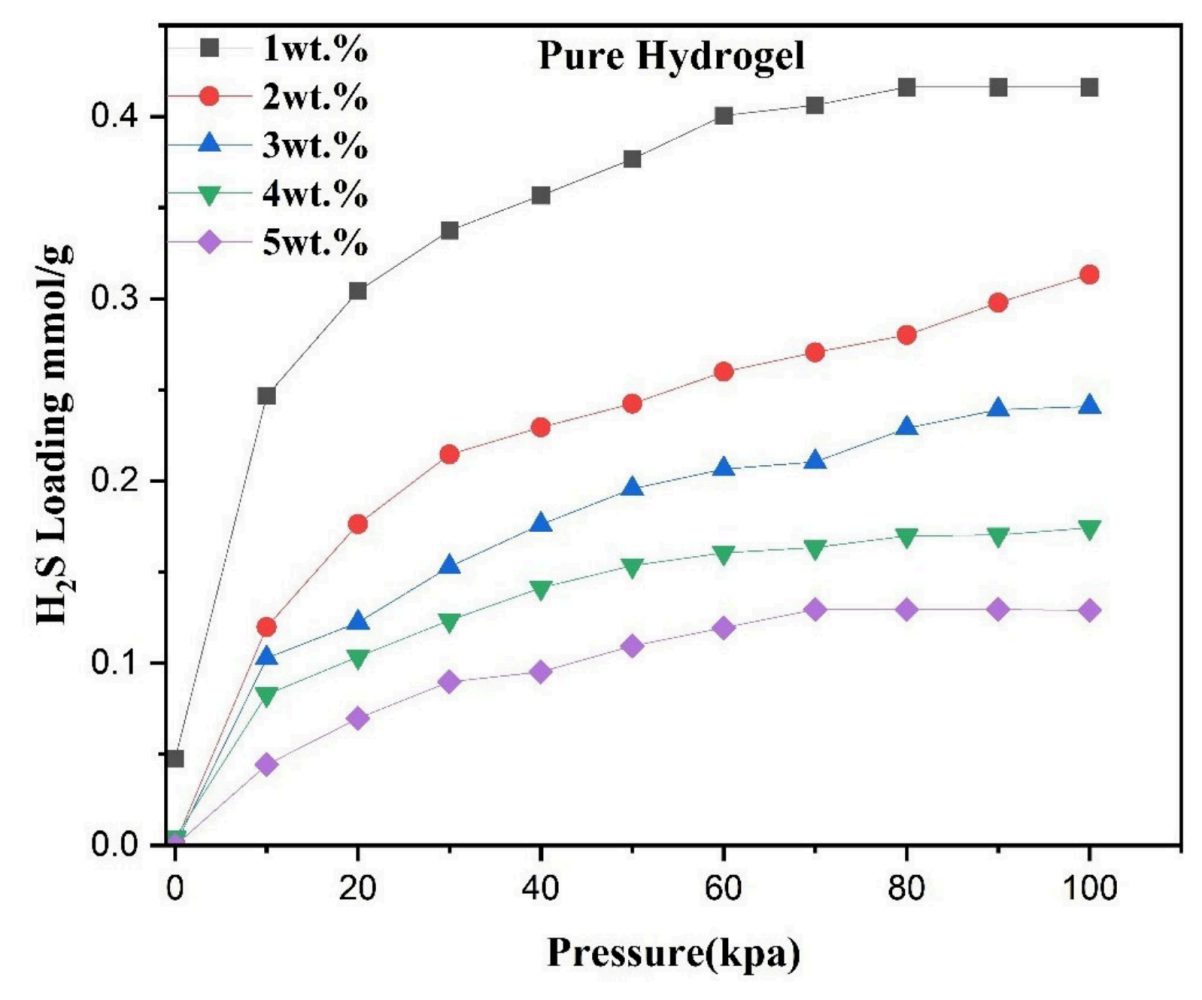
Simulated adsorption isotherm of H_2_S for pure PAA at different crosslinker concentrations.

**Figure 11:**
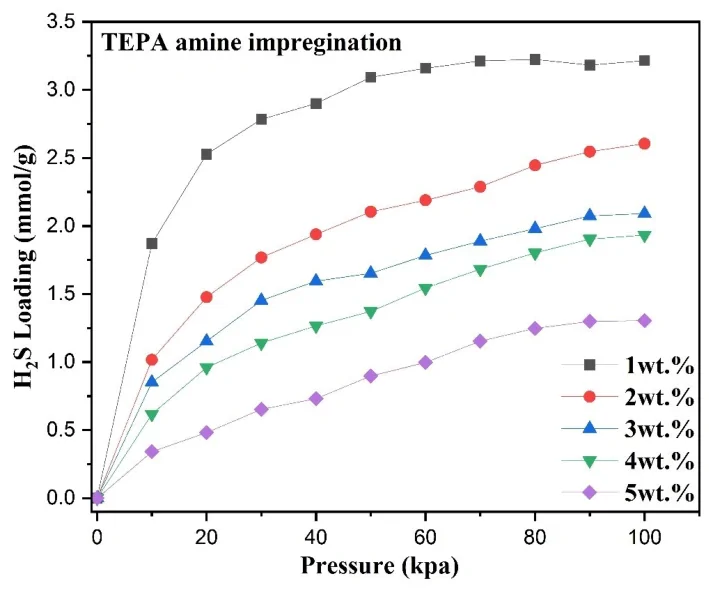
Adsorption isothermal study of H_2_S with TEPA amine impregnation.

The adsorption isotherms of H_2_S on pure PAA hydrogels with varying crosslinker ratios and TEPA amine impregnation were obtained through GCMC simulations using the adsorption isotherm task of the sorption module at 298 K, within a pressure range of 0.001 to 100 kPa. As shown in Figure 10, the results indicate that as the crosslinker content increases from 1 wt.% to 5 wt.%, the adsorption capacity of H_2_S decreases across the entire pressure range. The hydrogel with the lowest crosslinker content (1 wt.%) exhibits the highest H_2_S adsorption, reaching approximately 0.4 mmol/g at 100 kPa, while the hydrogel with the highest crosslinker content (5 wt.%) shows the lowest adsorption, remaining below 0.15 mmol/g. The observed decrease in adsorption capacity with increasing crosslinker content suggests that higher crosslinking reduces the free volume and available adsorption sites within the hydrogel matrix. Figure 11 presents the adsorption isotherms of H_2_S on TEPA-impregnated PAA hydrogels with different crosslinker ratios (1 wt.% to 5 wt.%) over a pressure range of 0–100 kPa. The results show a significant enhancement in H_2_S adsorption capacity compared to pure PAA hydrogels, demonstrating the strong affinity of TEPA amine groups for H_2_S molecules [45]. The hydrogel with the lowest crosslinker content (1 wt.%) exhibits the highest adsorption capacity, reaching approximately 3.0 mmol/g at 100 kPa, while the hydrogel with the highest crosslinker content (5 wt.%) shows the lowest capacity, remaining below 1.5 mmol/g. Previously, Sami et al. studied adsorption behaviors using the Grand Monte Carlo Simulation method in Materials Studio [46]. The significant increase in adsorption due to the high density of amine functional groups with TEPA impregnation was attributed to the strong chemical interactions between H_2_S and the amine functional groups.

## 4. Conclusions

In conclusion, adsorption behavior of PAA-based hydrogels was investigated before and after TEPA impregnation. FTIR analysis experimentally confirmed the characteristic functional groups of the synthesized hydrogels, while MD and GCMC simulations were used to study their molecular structure and H_2_S adsorption behavior. The simulations showed that TEPA impregnation enhanced the predicted H_2_S working capacity, reaching up to 3 mmol/g at 298 K compared with 0.4 mmol/g for the pure hydrogel. This improvement is attributed to the additional amine groups of TEPA, which provide favorable interaction sites for H_2_S. The MBA concentration also affected the polymer network structure and the accessibility of TEPA within the hydrogel. Overall, the results demonstrate the potential of TEPA-impregnated PAA hydrogels as polymer-based adsorbents for H_2_S removal, while the adsorption capacities reported in this study are computational predictions supported by the available FTIR characterization. Additionally, the crosslinker concentration was found to strongly influence the polymer network’s flexibility and the distribution of TEPA within the hydrogel matrix. Lower crosslinker concentrations resulted in a more open structure, enhancing the diffusion of H_2_S and maximizing the interaction with TEPA functional groups. This work provides valuable insights for the design of efficient polymer-based adsorbents for industrial gas purification.

## Data Availability

All data generated or analyzed during this study are included in this article and Supplementary Materials.

## Supplementary Materials

The following supporting information can be downloaded at: https://www.mdpi.com/article/10.3390/polym18202468/s1, Table S1: Calculation for H_2_S adsorption capacity using Equation (S4). References [47,48] are cited in the Supplementary Materials.

## Nomenclature

**Symbol**	**Description**
TEPA	Tetraethylenepentamine
PAA	Polyacrylic Acid
MBA	N′-methylene bis (acrylamide)
APS	Ammonium persulfate
EUV	Equilibrium uptake value
