# Supplementary material for: Effect of Crosslinker Concentration on Polyacrylic Acid Hydrogel for H2S Adsorption: Molecular Dynamic Simulation Study
Source: Polymers (Basel). 2026 Oct 9;18(20):2468. doi: 10.3390/polym18202468 (PMC13708947; doi:10.3390/polym18202468)
Supplement: Supplementary file 1 [file polymers-18-02468-s001.zip › polymers-4525736-supplementary.pdf]

S1- Calculation for H2S adsorption capacity (Behind the simulation)

The well known equation for the calculation of H2S adsorption capacity based on the breakthrough curve [1], [2] is given as

$$Q = \left(\frac{V}{22.4*W}\right)\int_0^t (C_{in} - C_{eff})dt \text{.........} \quad (S1)$$

$$Q = \left(\frac{V}{22.4*W}\right) C_{in} \int_0^t \left(1 - \frac{C_{eff}}{C_{in}}\right) dt \text{.........} \quad (S2)$$

Area under curve of the breakthrough curve:

$$t = \int_0^t \left(1 - \frac{C_{eff}}{C_{in}}\right) dt \text{.........} \quad (S3)$$

So, put equation (3) in equation (2)

$$Q = \left(\frac{V}{22.4*W}\right) A^* C_{in} \text{.........} \quad (S4)$$

where Q is the adsorbent's capacity of H_2S (mmol H_2S g^{-1}), t is the adsorption time (min), C_{in} and C_{eff} are the H_2S concentrations in the influent and effluent (vol fraction), respectively, V is the total flow rate (ml/min), W is the aerogel's weight (g), A is the area under curve of the breakthrough curve (min), and 22.4 is the molar volume of the gas (ml $mmol^{-1}$).

Table. S1. Calculation for H_2S adsorption capacity using equation (4)

**Sample (Crosslinker ratio)**	**Q (g/g)**
**1.wt%**	**5.74**
**2.wt%**	**4.43**
**3.wt%**	**3.21**
**4.wt%**	**2.5**
**5.wt%**	**2.2**

REFERENCES

[1] X. Wang, J. Liu, W. Liu, Y. Gong, and Y. Sun, "Amine-impregnated polymeric resin with high CO_2 adsorption capacity for biogas upgrading," *Chem. Eng. J.*, vol. 430, no. P2, p. 132899, 2021, doi: 10.1016/j.cej.2021.132899.

[2] Y. Chen, J. Zhang, H. Liu, X. Wang, and S. Chen, "A surfactant modified solid amine adsorbent to enhance CO_2 adsorption performance," *Colloids Surfaces A Physicochem. Eng. Asp.*, vol. 677, no. PA, p. 132323, 2023, doi: 10.1016/j.colsurfa.2023.132323.
